# A Case of Preserved Blood Flow to the Portal Vein Due to the Concurrent Reconstruction of the Superior Mesenteric Vein and the Splenic Vein Using an Artificial Blood Vessel

**DOI:** 10.7759/cureus.31457

**Published:** 2022-11-13

**Authors:** Masahiro Takeuchi, Masahiko Onoda, Michinori Iwamura, Toshihiro Inokuchi, Kazuaki Kawano, Tomoe Katoh, Akira Furutani

**Affiliations:** 1 Department of Surgery, Yamaguchi Rosai Hospital, Sanyo-onoda, JPN

**Keywords:** eptfe, artificial blood vessel, splenic vein, portal vein resection, pancreatic head cancer

## Abstract

Pancreatic cancer is often advanced and invades the major blood vessels around the pancreas. Portal vein (PV) and/or superior mesenteric vein (SMV) resection is performed for radical resection. In such cases, end-to-end anastomosis is best if the remnant vein is sufficiently long. However, when the excision distance is long, reconstruction requires an artificial blood vessel. In contrast, there is no consensus concerning the need for splenic vein (SV) reconstruction. We herein report a case in which portal vein thrombus and congestion of the bowel that occurred after PV-SMV reconstruction were improved by additional anastomosis of the PV-SV.

## Introduction

Pancreaticoduodenectomy with resection of the splenoportal junction (SPJ) is often performed for advanced pancreatic cancer. In gastroenterological surgery, revascularization is performed via end-to-end anastomosis whenever possible, as the surgical field is contaminated and there are risks of rupture, obstruction, and infection.

Approaches for vein reconstruction with artificial blood vessels have not been completely established, so portal vein (PV) reconstruction is performed by an end-to-end anastomosis with approximately 5.0-cm resection of the SPJ [1,2]. However, in cases with defects >7.0 cm, reconstruction with an artificial blood vessel is required. In such cases, anastomosis of the splenic vein (SV) is not usually a serious problem.

We herein report a case in which the PV-superior mesenteric vein (SMV) was reconstructed using an artificial blood vessel after pancreaticoduodenectomy for pancreatic cancer but caused PV thrombus and congestion of the bowel, which was improved by an additional anastomosis of the PV-SV.

## Case presentation

A 76-year-old man was referred to our hospital for resection of pancreatic head cancer with obstructive jaundice. He has diabetes treated with insulin (HbA1c: 8.8%) and untreated nonalcoholic steatohepatitis (Child-Pugh A). Laboratory data showed jaundice (total bilirubin: 8.9 mg/dl, direct bilirubin: 7.1 mg/dl), liver injury (aspartate aminotransferase/alanine aminotransferase: 193/297 IU/l, alkaline phosphatase: 1382 IU/l, γ-glutamyl transpeptidase: 851 IU/l) and abnormal tumor marker levels (carcinoembryonic antigen: 7.2 ng/ml, DUPAN-2: 6356 U/ml).

**Figure 1:**
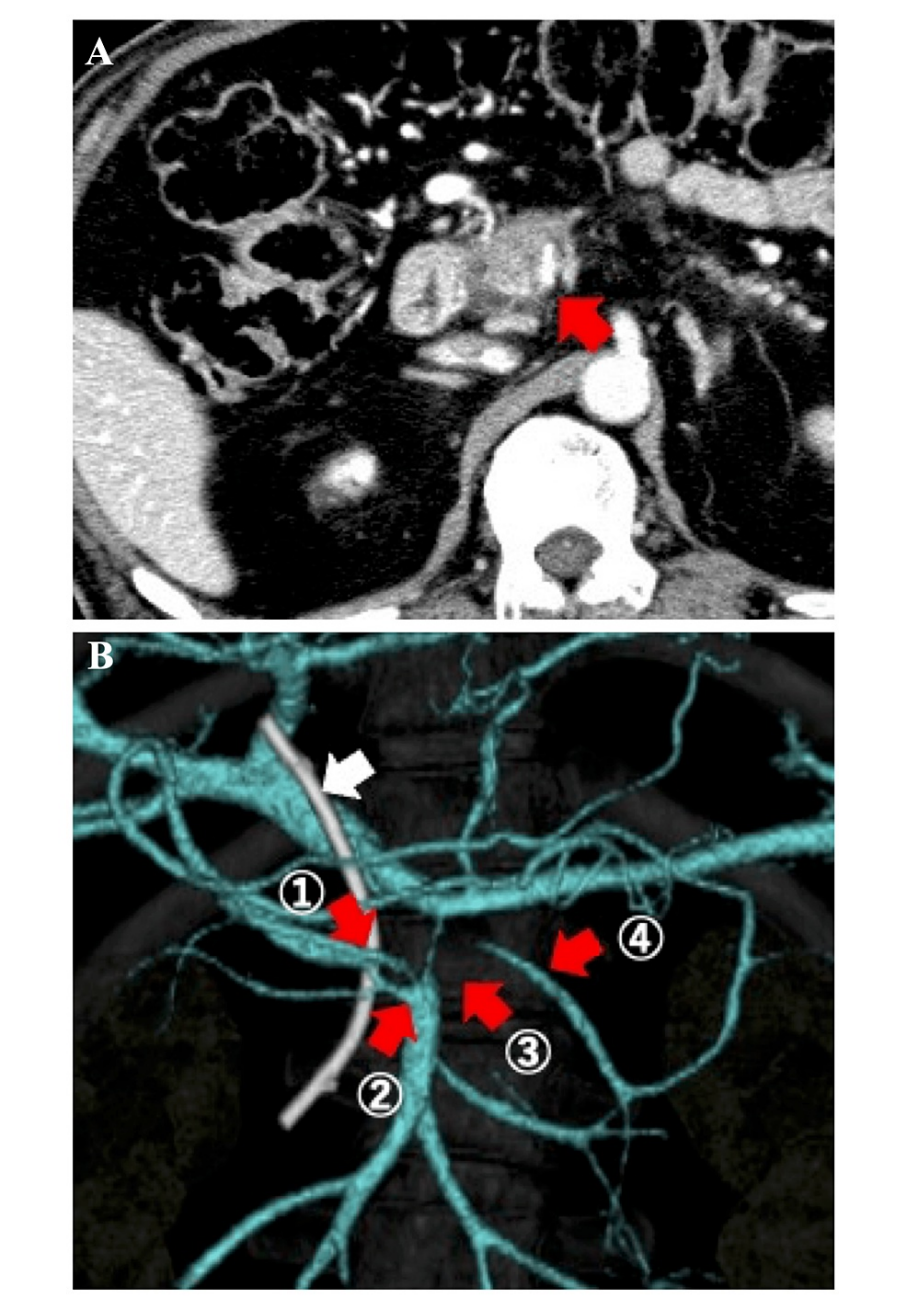
Computed tomography (CT) and three-dimensional CT findings (A) Tumor of 3.0 cm located in the pancreatic head and suspected superior mesenteric vein (SMV) invasion (red arrow). (B) Stenosis by tumor invasion. The red arrow indicates the ① middle colic vein, ② gastrocolic trunk, ③ superior mesenteric vein, and ④ inferior mesenteric vein. The white arrow indicates the endoscopic retrograde biliary drainage tube.

Computed tomography (CT) revealed a tumor 3.0 cm in size located in the pancreatic head with no distant metastasis (Figure 1A). Three-dimensional CT findings suggested an invasion of the PV (Figure 1B). Based on these findings, we made a diagnosis of borderline resectable pancreatic cancer (T3N0M0) [3].

**Figure 2:**
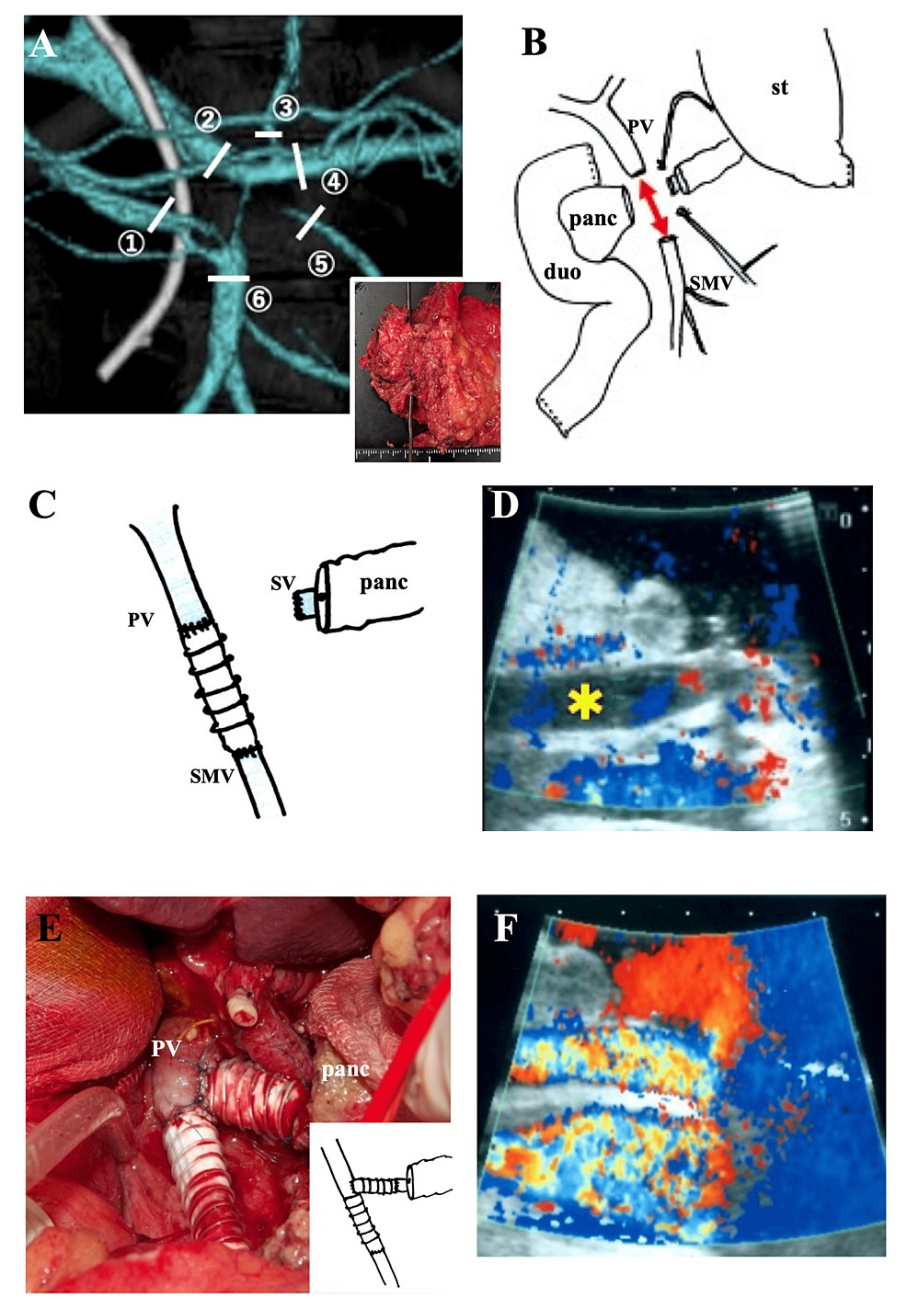
Surgical findings (A) Combined resection of the involved vein by the tumor. The white bar indicates the ① middle colic vein, ② portal vein, ③gastrocoronary vein, ④ splenic vein, ⑤ inferior mesenteric vein, and ⑥ superior mesenteric vein. The sonde is implanted in the PV and the tumor has invaded the PV. (B) After resection of the tumor and veins. The red arrow shows the defect length from the portal vein to the superior mesenteric vein (7.0 cm). (C) Initial revascularization. PV-SMV anastomosis. (D) Ultrasonography of the portal vein when the bowel was congested. The yellow asterisk indicates thrombosis. (E) Additional PV-SV anastomosis. (F) Improvement of the blood flow in the PV. st: stomach, PV: portal vein, SMV: superior mesenteric vein, SV: splenic vein, panc: pancreas, duo: duodenum

We achieved complete resection (pylorus-preserving pancreatoduodenectomy) of the pancreatic head cancer after improving obstructive jaundice [4]. The main invasion of the veins was in the SPJ, reaching 7.0 cm (Figures 2A, 2B). Revascularization was initially achieved via anastomosis of the PV-SMV through a ringed expanded polytetrafluoroethylene (ePTFE) graft with non-absorbable suture material (Medtronic Surgipro, 5-0; Medtronic plc, Minneapolis, Minnesota) for 30 minutes after clamping (Figure 2C). However, the congestion of the intestine was not improved, and thrombosis was identified in the PV (Figure 2D). The thrombosis was immediately removed with a Fogarty balloon catheter. To prevent thrombosis formation due to the low blood flow in the PV, a PV-SV anastomosis was added with a ringed ePTFE graft of a 3.0cm length (Figure 2E). The congestion of the intestine and blood flow in the PV were subsequently improved (Figure 2F).

**Figure 3:**
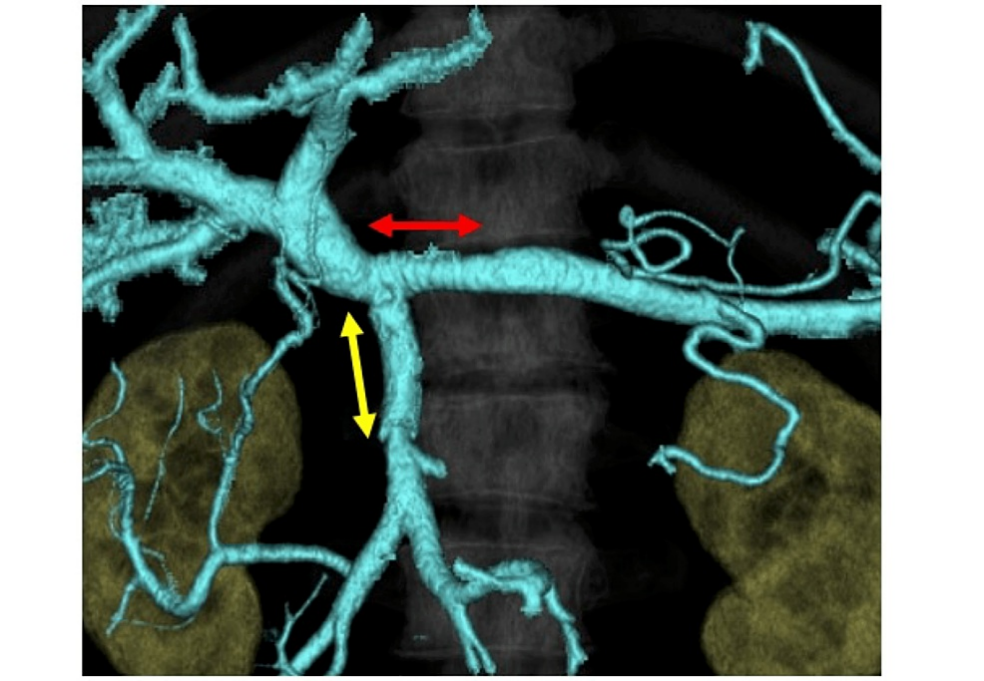
Three-dimensional CT findings after surgery (two months later) Red and yellow arrows indicate anastomosis of the PV-SV and PV-SMV, respectively.

Aspirin and warfarin were orally administered daily, starting after five days from the surgery for anti-coagulation. The patient was discharged on postoperative day 16 without any major complications, and two months later, the grafts showed good patency on a CT scan (Figure 3).

## Discussion

Pancreatic cancer is one of the most lethal malignant tumors worldwide [5]. Approximately 65% of pancreatic cancers occur in the head, showing a poor prognosis compared with cancer in other areas of the pancreas. Most pancreatic cancer patients are commonly diagnosed in their advanced stage, and only 20% are operable [6].

In the past, the presence of vascular involvement prompted the consideration of radical resection or palliative surgery. At present, patients with PV invasion have the opportunity to be cured thanks to marked improvements in techniques and perioperative management. However, to undergo reconstruction of the PV-SMV, cases must meet certain criteria. For cases with blood vessel invasion accounting for <1/3 of the circumference and with less severe involvement, sutures and patches are used. For cases with invaded blood vessels ≥1/3 of the circumference and <5 cm in size, venous end-to-end anastomosis is used. For cases with invaded blood vessels ≥5 cm in size, artificial blood vessels are commonly used. However, the determination of the severity of the invaded PV is left to the intraoperative judgment of the operators [1].

In the present case, artificial blood vessels were used because there was a 7.0-cm defect after PV resection. Furthermore, the autogenous vein (end-to-end anastomosis, sutures, and patches) is more likely to cause increased tension in the anastomosed PV, reflected in stenosis, which may thereafter induce portal hypertension. A ringed ePTFE can reduce these risks. Compliance, lack of thrombogenicity, and resistance to infections as well as the ability to heal, remodel, contract, and secrete normal blood vessel products are theoretical advantages of artificial blood vessels [7,8]. The ringed ePTFE graft is considered to have strong resistance to infection compared with other artificial vascular grafts. When using a ringed ePTFE graft in liver surgery, no graft infection has yet been reported [9]. Furthermore, in addition, compared with the autogenous vein, ringed ePTFE grafts show very high patency rates and resistance to graft collapse, resulting in few cases of graft thrombosis [10]. We initially performed reconstruction with a ringed ePTFE graft for PV-SMV. However, the bowel showed congestion due to induced thrombosis in the PV.

Cases with PV involvement frequently show obstructive jaundice. Cholangitis causes inflammation around the PV and induces thrombus due to increasing coagulation ability [11,12]. In the present case, who had cholangitis due to obstructive jaundice, some preoperative factors may have affected the occurrence of PV thrombosis. There is still no consensus regarding the best method for performing reconstruction of the PV-SV after PV resection in the SPJ area, but some reports have described the occurrence of portal hypertension involving organs on the left side (esophageal/gastric varix, splenomegaly) without SV reconstruction [13]. The SV has an antegrade flow to the PV. In the present case, the PV-SV was added to prevent thrombosis by increasing the blood flow in the PV. In cases that are preoperatively confirmed to have thrombotic factors, PV-SV should be positively considered.

## Conclusions

Pancreaticoduodenectomy combined with PV resection and reconstruction with artificial blood vessels is a safe and appropriate therapy choice for respectable advanced pancreatic head cancer patients with PV involvement. We encountered a case wherein the blood flow to the PV was preserved due to the reconstruction of the SMV and SV using ringed ePTFE grafts at the same time.
